# *In Silico* Identification of Type III PKS Chalcone and Stilbene Synthase Homologs in Marine Photosynthetic Organisms

**DOI:** 10.3390/biology9050110

**Published:** 2020-05-22

**Authors:** Daniele De Luca, Chiara Lauritano

**Affiliations:** 1 Department of Humanities, Università degli Studi Suor Orsola Benincasa, CAP80135 Naples, Italy; 2 Department of Marine Biotechnology, Stazione Zoologica Anton Dohrn, CAP80121 Naples, Italy

**Keywords:** microalgae, type III polyketide synthases (PKS), chalcone synthase, stilbene synthase, transcriptome analysis

## Abstract

Marine microalgae are photosynthetic microorganisms at the base of the marine food webs. They are characterized by huge taxonomic and metabolic diversity and several species have been shown to have bioactivities useful for the treatment of human pathologies. However, the compounds and the metabolic pathways responsible for bioactive compound synthesis are often still unknown. In this study, we aimed at analysing the microalgal transcriptomes available in the Marine Microbial Eukaryotic Transcriptome Sequencing Project (MMETSP) database for an *in silico* search of polyketide synthase type III homologs and, in particular, chalcone synthase (CHS) and stilbene synthase (STS), which are often referred to as the CHS/STS family. These enzymes were selected because they are known to produce compounds with biological properties useful for human health, such as cancer chemopreventive, anti-inflammatory, antioxidant, anti-angiogenic, anti-viral and anti-diabetic. In addition, we also searched for 4-Coumarate: CoA ligase, an upstream enzyme in the synthesis of chalcones and stilbenes. This study reports for the first time the occurrence of these enzymes in specific microalgal taxa, confirming the importance for microalgae of these pathways and giving new insights into microalgal physiology and possible biotechnological applications for the production of bioactive compounds.

## 1. Introduction

Microalgae are photosynthetic organisms adapted to live in several different environments, including marine, freshwater, polar, temperate and tropical. They are also known to produce a plethora of metabolites derived from primary and secondary metabolism, which have shown possible applications for human health (e.g., compounds with anti-cancer, anti-microbial, anti-diabetes, anti-epilepsy, anti-inflammatory, anti-atherosclerosis, anti-osteoporosis, immunomodulatory and antioxidant activities; [1,2,3,4,5,6,7,8,9,10]). Various studies have focused on the discovery of metabolic pathways involved in the synthesis of bioactive compounds [11,12,13,14]. Even if microalgae can be cultured in big volumes to get large amounts of the compounds of interest, heterologous expression of the enzyme responsible for the compound synthesis and its production in a host can be a valuable alternative for meeting industrial demands.

**Figure 1:**
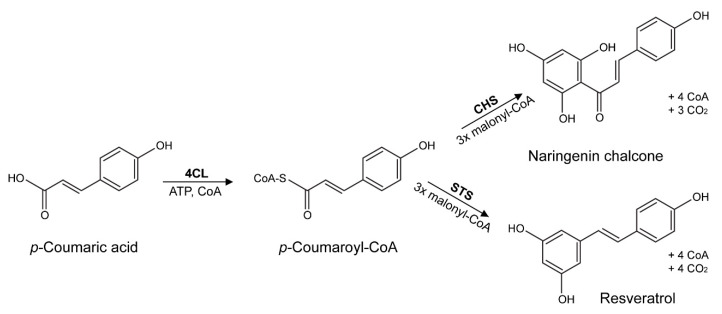
Enzymatic reactions catalysed by type III PKSs for the production of naringenin chalcone and resveratrol.

Type III polyketide synthases are a group of enzymes present in bacteria, fungi and plants [15,16,17,18], specialized in the production of important secondary metabolites (aromatic polyketides) with possible pharmaceutical, nutraceutical and cosmeceutical applications [8,11] of increasing economic interest [19]. These compounds are produced by the sequential decarboxylative addition of three acetate units from malonyl-CoA to a p-coumaryl-CoA starter molecule without the involvement of acyl carrier proteins (ACPs) [19,20] (Figure 1). Chalcone synthase (CHS; EC 2.3.1.74) and stilbene synthase (STS; EC 2.3.1.95) are probably the best studied enzymes of this group, which is often referred to as the CHS/STS family [21]. These two enzymes share 75–90% amino acid sequence identity over their ~400 residues [15,22] and use the same substrates (*p*-Coumaroyl-CoA and malonyl-CoA) and basic mechanism of catabolism [19]. However, each enzyme yields different products—naringenin chalcone (CHS) and resveratrol (STS) respectively—which differ in the atoms involved in the closure of ring structures [23]. Furthermore, the typical STS reaction includes a decarboxylation step that is not performed by CHS [24]. Naringenin chalcone is the precursor of many polyphenolic compounds, such as anthocyanins, chalcones, flavanones, dihyroflavonols, flavans, flavones, flavonols and isoflavonoids [25,26], and it is found in considerable quantities in *Citrus* fruits (lemon, lime, mandarin, orange), potatoes and tomatoes [27,28]. On the contrary, resveratrol is a polyphenolic compound (phytoalexin) that is only found in several unrelated plant families containing the stilbene synthase gene, such as Vitaceae, Dipterocarpaceae, Ericaceae, Leguminosae, Pinaceae and Polygonaceae [29,30,31,32,33,34], and in various foods and food products (e.g., berries, wines, juices) derived from them [35,36,37].

Chalcone synthase plays a key role in the synthesis of several metabolites (mostly flavonoids) that work as floral pigments, antibiotics, UV protectants and insect repellents [38,39,40]. Flavonoids are also useful for human health, working as cancer chemopreventive [41,42,43], antioxidant [44,45,46], anti-asthmatic [47,48,49], anti-inflammatory [50,51,52], anti-microbial [53,54,55] and anti-malarial [56,57,58] agents. Similarly, resveratrol, produced by the enzyme stilbene synthase, has proven to be a potent anti-angiogenic [59,60,61], anti-diabetic [62,63,64], anti-cancer [65,66,67], anti-viral [68,69,70], cardioprotective [71,72,73] and neuroprotective [74,75,76] compound, as well as a more powerful antioxidant and vasorelaxant than naringenin [77,78,79].

From an evolutionary point of view, STS protein sequences do not form a cluster of their own but are grouped with the CHS sequences of the same or related organisms rather than with other STS [23,24,80]. This suggested the hypothesis that there was no ancestral STS gene and that STS genes developed from CHS recurrently and independently [24]. The latter hypothesis is supported by the fact that, currently, STS genes have been isolated from a small number of unrelated higher plants (see above) and that in vitro studies have shown that few amino acid changes are sufficient to convert a CHS into a protein with STS activity [24,81]. Even if most of the known chalcone and stilbene synthase genes have been isolated from gymnosperms and angiosperms, there are also reports from other land plant lineages. For instance, CHS-like genes were found in ferns [82] and liverworts [83]. The occurrence of homologs of some of the land plant flavonoid/stilbenoid pathway genes in other lineages of marine photosynthetic organisms is still controversial. No type III PKS genes have been found in various microalgal genomes, such as of the diatoms *Phaeodactylum tricornutum* and *Thalassiosira pseudonana*, or in red algae [84]. This led to the conclusion that these genes have probably been acquired after the land conquest, possibly by lateral gene transfer from bacteria or fungi [84]. However, recent studies have confirmed the presence of putative type III PKS enzymes in the brown alga *Ectocarpus siliculosus* [84] and the dichtyochophyte *Pseudochattonella farcimen* [85] and of some genes involved in the phenylpropanoid pathway in streptophyte algae [86]. On the basis of these new data, it was hypothesised that the lateral gene transfer event of type III PKS genes must have occurred after the separation of diatoms from other ochrophytes, but before the divergence of brown algae with pelagophytes and dichtyochophytes [84]. To date, only a few nuclear genomes are available for microalgae to corroborate such assumptions, mostly due to their large sizes and high complexity, especially for dinoflagellates [87,88], which make them difficult to analyse. As a consequence, the study of gene function and metabolic pathways in these microorganisms has been mostly done through transcriptome sequencing [11,89,90,91,92]. Indeed, targeting only expressed coding regions, transcriptome sequencing overcome the issues associated to sequencing and assembly of introns, intergenic and repetitive regions common to eukaryotes [93]. Furthermore, thanks to the Marine Microbial Eukaryotic Transcriptome Sequencing Project (MMETSP), hundreds of transcriptomes from the most abundant and ecologically significant microbial eukaryotes in the oceans have been made available to the public [93].

In this study, we aimed at shedding light on the possible occurrence of chalcone and stilbene synthase genes in marine photosynthetic eukaryotes, with special regard to STS due to relevant pharmaceutical activities of resveratrol. We searched for CHS/STS homologs in the transcriptomes of photosynthetic marine organisms from MMETSP [93]. We annotated the sequences retrieved from BLAST search and inferred phylogenetic trees. To further confirm the validity of our findings, we also searched for homologs of 4-Coumarate: CoA ligase (4CL), an upstream enzyme in flavonoid and stilbenoid biosynthesis in land plants. 

## 2. Materials and Methods

### 2.1. Identification and Annotation of CHS/STS and 4CL Homologs

The STS protein sequence of the common grape wine *Vitis vinifera* (acc. numb. P28343) was used as a query for a BLASTP [94] search of CHS/STS homologs, whereas the 4CL1 sequence of *Arabidopsis thaliana* (acc. numb. NP_175579) was used for the 4-Coumarate: CoA ligase homolog search. These two sequences were used as queries for a BLASTP [94] search against the 103 transcriptomes of marine photosynthetic organisms from the MMETSP Project (https://zenodo.org/record/12125852585; Table S1). The BLASTP analyses were conducted, setting a threshold of homology to 1E-10. The retrieved homolog sequences were functionally annotated using the eggNOG mapper (http://eggnog-mapper.embl.de/) [95,96]. Transcripts annotated as “chalcone stilbene synthases family”, “naringenin-chalcone synthase activity” and “PFAM chalcone and stilbene” for CHS/STS, and “4-coumarate-CoA”, “4-coumarate-CoA ligase” and “4-coumarate-CoA ligase-like” for 4CL were considered valid and used for this study.

### 2.2. Sequence Alignment, Trimming and Phylogenetic Inference

To ascertain the evolutionary relationships among CHS/STS genes of marine photosynthetic organisms and the well-studied terrestrial counterpart (land plants), we included in our analysis the sequences of STS, CHS and CHS-like genes of representative organisms from different taxonomic categories (Table S2). As outgroups, we used the sequences of 3-ketoacyl-CoA synthase (KCS) from representative taxa (Table S2), which have previously proven to be the sister group of CHS/STSs in the thiolase superfamily [80]. For 4CL, we downloaded from the GenBank ingroup and outgroup sequences from different taxa (Table S2) and used the sequences of luciferases as outgroup taxa [97].

Sequences were aligned using COBALT [98] (available at https://www.ncbi.nlm.nih.gov/tools/cobalt/). This software uses sequence information together with protein-motif regular expressions (PROSITE database) and conserved protein domains (NCBI CDD database) to produce biologically meaningful multiple alignments [98]. Poorly aligned regions were removed with trimAl v1.2 [99] using the *automated1* option to find the most appropriate mode to trim the alignment (use of gaps or similarity scores) depending on the alignment characteristics. A maximum likelihood phylogenetic tree was inferred for both CHS/STS and 4CL genes in PhyML [100] using the evolution model suggested by Smart Model Selection (SMS) [101]. Support to nodes was calculated using the Shimodaira-Hasegawa-like (aLRT SH-like) procedure [102]. The resulting tree was visualised and graphically edited in FigTree v1.4.3 (http://tree.bio.ed.ac.uk/software/figtree/).

### 2.3. Protein Domain Assignment

The microalgal strains exhibiting multiple CHS/STS homologs after functional annotation and phylogenetic inference were further analysed to annotate the conserved domains within the protein sequence using InterProScan 5 [103]. For each protein sequence, we reported the occurrence of N and C-terminus domains, which are relevant to biochemical activity [104] and their localisation along the transcript.

## 3. Results

### 3.1. Retrieval, Annotation and Phylogenetic Analysis of STS/CHS Homologs

**Figure 2:**
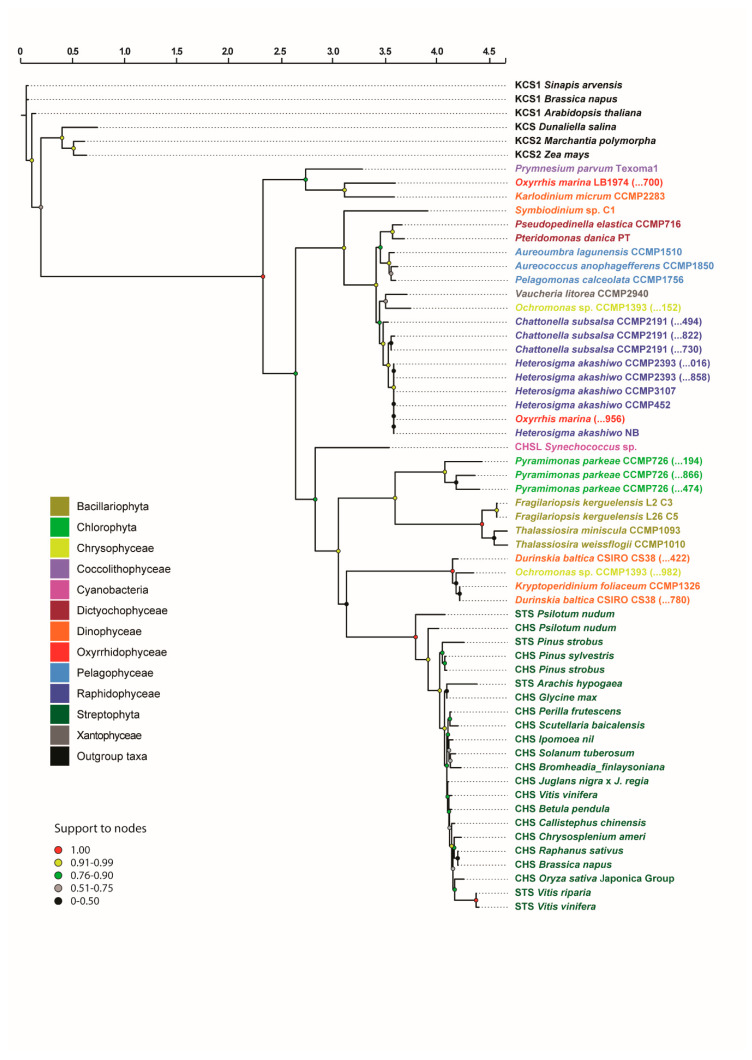
Maximum likelihood phylogenetic tree of chalcone/stilbene synthases. Numbers in parentheses after strain denomination refer to the last three codes of transcripts. Support to nodes was inferred using the Shimodaira-Hasegawa-like test.

The BLASTP search against the 112 transcriptomes of MMETSP (103 of photosynthetic microorganisms plus 9 of not-photosynthetic ones) returned 43 homolog sequences in 27 taxa (Table S1). After functional annotation, 31 homologs in 24 taxa were left (Table S1, File S1). According to the results, the functional filter allowed the removal of protein sequences with conserved domains but different function in the same organisms, since 12 sequences and only 3 taxa were lost. In total, the homologous sequences used for phylogenetic inference (including the ones from GenBank) corresponded to 12 classes/phyla: Bacillariophyta, Chlorophyta, Chrysophyceae, Coccolithophyceae, Cyanobacteria, Dictyocophyceae, Dinophyceae, Oxyrrhidophyceae, Pelagophyceae, Raphidophyceae, Streptophyta and Xantophyceae (Figure 2).

The final alignment (after trimming procedure) included 60 sequences and 249 characters (File S2). The best evolutionary model for the protein alignment selected using the Akaike Information Criterion (AIC) was the LG+G+I model [105]. The maximum likelihood phylogenetic tree of CHS/STS genes (Figure 2) confirmed that all retrieved and annotated homologs form a monophyletic group that is sister to 3-ketoacyl-CoA synthases (KCSs), the latter considered the closest outgroup within the thiolase superfamily [80]. Within this group, a first, highly supported (1.00 support value) branching event led to the separation of CHS/STS-like genes of *Prymnesium parvum* Texoma1 (Coccolithophyceae), *Oxyrrhis marina* LB1974 (Oxyrrhidophyceae) and *Karlodinium micrum* CCMP2283 (Dinophyceae) from all other sequences. The next branching event in the phylogenetic tree (0.76–0.90 support value) separates two main clades of CHS/STS- like sequences—one containing sequences of Dictyocophyceae, Pelagophyceae, Raphidophyceae and Xantophyceae, with the sequence of *Symbiodinium* sp. C1 as sister taxon, and another one encompassing sequences of diatoms (Bacillariophyta), green algae (Chlorophyta), dinoflagellates (Dinophyta), chrysophyceae and the land plants (Streptophyta) (Figure 2). The sequence of a CHS-like gene belonging to the cyanobacterium *Synechococcus* sp. is sister to this clade. The localisation of one of the two sequences found in the transcriptome of the chrysophyte *Ochromonas* sp. CCMP1393 in this clade next to other dinoflagellates (despite with low support) is likely to constitute an artefact due to its shorter length (less than half) in respect to other sequences in the alignment (File S1). The CHS/STS sequences of land plants formed, as expected, a highly supported (1.00) clade, in which the sequences of CHS and STS do not form a separate cluster but are grouped together.

### 3.2. Retrieval, Annotation and Phylogenetic Analysis of 4CL Homologs

Homologs for 4-Coumarate: CoA ligase (4CL) were found in 106 out of 112 transcriptomes analysed (including non-photosynthetic organisms). However, after functional annotation, only 24 sequences in 22 transcriptomes (species) were left (Table S1, File S3). Most of the sequences were indeed annotated as “2-succinylbenzoate-CoA ligase”, “Acetyl-coenzyme A synthetase” or “AMP-binding enzyme”. Two homologs were found in the transcriptome of the dinoflagellates *Karenia brevis* SP1 and *Kryptoperidinium foliaceum* CCMP1326 each (File S3).

**Figure 3:**
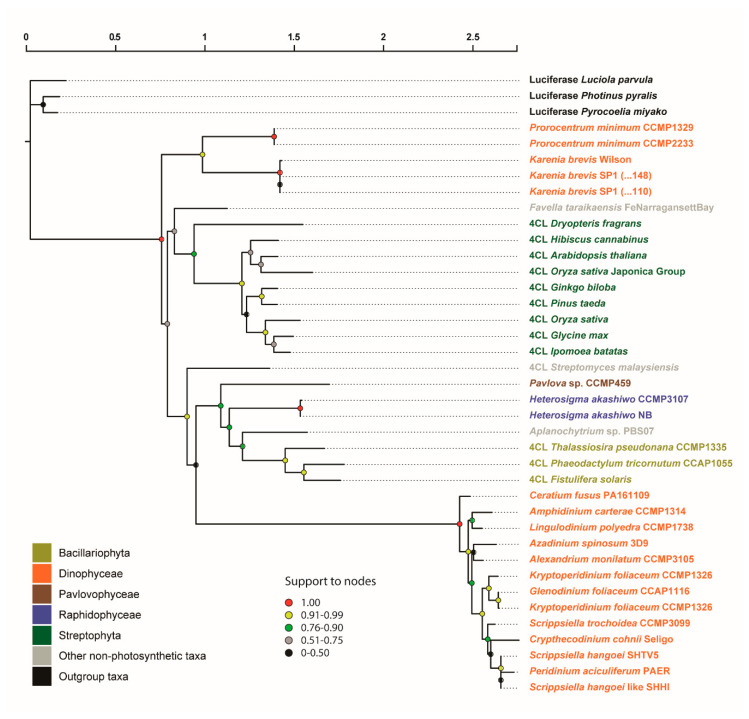
Maximum likelihood phylogenetic tree of 4-Coumarate:CoA ligase. Numbers in parentheses after strain denomination refer to the last three codes of transcripts. Support to nodes was inferred using the Shimodaira-Hasegawa-like test.

The final alignment (after removal of poorly aligned regions) consisted of 317 aa (File S4). The best evolutionary model of protein evolution selected using the AIC was the LG+G+F [105]. The inferred phylogenetic tree showed that the sequences of the dinoflagellates *Karenia brevis* SP1 and *Kryptoperidinium foliaceum* CCMP1326 formed a highly supported clade (support > 0.91) that was separated from all the other sequences (1.00 support value) (Figure 3). Within this latter group, the sequences of marine photosynthetic microorganisms (Bacillariophyta, Pavlovophyceae, Raphidophyceae) formed a monophyletic group (support value > 0.75) sister to the 4CL sequence of the actinobacterium *Streptomyces malaysiensis* (support > 0.91). Dinoflagellate sequences form a highly supported clade (1.00) but the phylogenetic relationships with neighbour taxa are not resolved (support value < 0.50). The sequences of land plants formed a monophyletic group, with the sequences of gymnosperms and angiosperms closely related (support > 0.91) and of the fern *Dryopteris fragrans* as sister group (support > 0.75). The sequence of the ciliate *Favella taraikaensis* is found close to Streptophyta, but the support was low (0.51–0.75).

### 3.3. Identification of Taxa with CHS/STS- and 4CL-Like Enzymes

Our transcriptome survey revealed that after functional annotation, homologs for both CHS/STS and 4CL genes were only found in the raphidophyte *Heterosigma akashiwo* strains CCMP3107 and NB, and the dinoflagellate *Kryptoperidinium foliaceum* (Dinophyceae) strain CCMP1326 (Table S1). Other strains of *Heterosigma akashiwo* failed the annotation step for 4CL gene (Table S1). However, in many other taxa (e.g., other dinoflagellates, diatoms, dictyocophyceae, pelagophyceae, chrysophyceae and oxyrrhidophyceae) we found both CHS/STS and 4CL homologs, but the latter were not functionally recognised as 4-Coumarate: CoA ligases (Table S1).

At a higher taxonomic level, homologs for both genes were mostly found in Bacillariophyta, Dinophyceae and Raphidophyceae. In the trancriptomes of *Pyramimonas parkeae* CCMP726 and *Chattonella subsalsa* CCMP2191 we found three homologs corresponding to CHS/STS-like genes each, with different degree of similarity. In the chlorophyte *Pyramimonas parkeae* CCMP726, these homologs shared a homology between 53–63%, whilst in the case of the raphidophyte *Chattonella subsalsa* CCMP2191 homology was between 89–96%. 

### 3.4. Protein Domain Assignment

**Figure 4:**
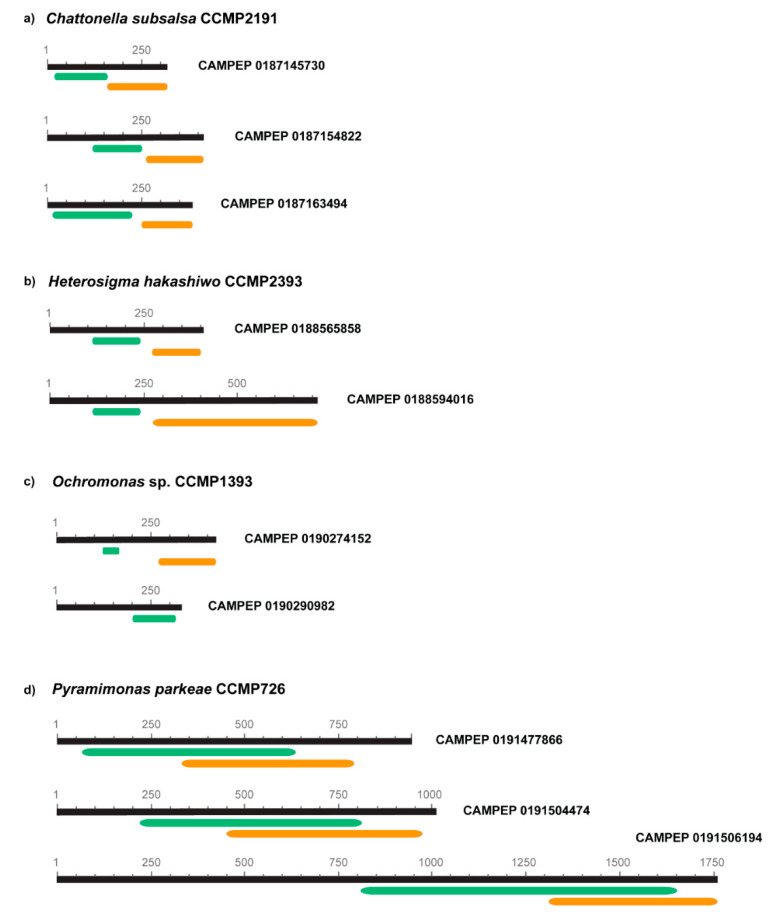
Localisation and annotation of chalcone/stilbene synthase domains in strains containing multiple transcripts: *Chattonella subsalsa* CCMP2191 (**a**), *Heterosigma akashiwo* CCMP2393 (**b**), *Ochromas* sp. CCMP1393 (**c**), and *Pyramimonas parkeae* CCMP726 (**d**). Green bars refer to N-terminus domains, whereas orange bars to C-terminus ones. Transcript codes (CAMPEP) are as in File S1.

Four microalgal strains, *Chattonella subsalsa* CCMP2191, *Heterosigma akashiwo* CCMP2393, *Ochromonas* sp. CCMP1393 and *Pyramimonas parkeae* CCMP726, contained more than one CHS/STS transcript. The domain analysis revealed that all of these sequences contain the CHS/STS domains, both at N terminus (IPR001099, green bars) and C terminus (IPR012328, orange bars) (Figure 4). The only exception was the CAMPEP 0190290982 transcript of *Ochromonas* sp. CCMP1393, which contained only a portion of the CHS/STS domain of around 100 aa at the N terminus (Figure 4c). This might result in an artefact, also explaining why this sequence is misplaced in the phylogenetic tree, close to dinoflagellates instead of other phylogenetically related taxa as well as the other transcript from the same species (Figure 2). Furthermore, this transcript is likely to be non-functional, because of the lack of the C-terminus domain. The three CHS/STS transcripts found in *Pyramimonas parkeae* CCMP726 presented an overlapping match of N and C domains (Figure 4d). This could be interpreted as either the result of complex structural gene rearrangements that occurred during the evolutionary history of the species or contrasting predictions of domain architecture in InterPro.

## 4. Discussion

Type III polyketide synthases were initially thought to be plant-exclusive enzymes with a pivotal role in the biosynthesis of flavonoids and several secondary metabolites as chalcones, stilbenes, benzophenones, acridones, phloro-glucinols and resorcinols [16,22]. Subsequently, these enzymes were also found in some bacteria [15], fungi [18] and brown algae [84]. Several transcriptome surveys have demonstrated that marine protists may encode several PKS enzymes, especially of type I and II [106,107,108,109]. However, to date, proper knowledge of the occurrence and function of type III PKS enzymes in marine photosynthetic organisms is still lacking. 

In this paper, we focused our attention on a specific class of type III PKSs, the chalcone family, which includes enzymes involved in the production of chalcones and stilbenes (e.g., resveratrol) and their precursor enzyme 4-Coumarate: CoA ligase (4CL). Our transcriptomic search reveals the occurrence of CHS/STS-like genes in several lineages of marine photosynthetic microorganisms (Bacillariophyta, Chlorophyta, Chrysophyceae, Coccolithophyceae, Dictyochophyceae, Dinophyceae, Oxyrrhidophyceae, Pelagophyceae, Raphidophyceae and Xantophyceae). From the phylogenetic point of view, the sequences of diatoms (*Fragilariopsis kerguelensis*, *Thalassiosira miniscula* and *Thalassiosira weissflogii*) and some dinoflagellates (*Durinskia baltica* and *Kryptoperidinium foliaceum*), as well as the ones of the green alga *Pyramimonas parkeae* could be considered as the most likely CHS/STS-like candidates (Figure 2). This is because they are in the same, highly supported clade that contains the true CHS/STS genes of land plants (Streptophyta) and is sister to the cyanobacterium *Synechococcus*. The sequences of Dictyochophyceae, Pelagophyceae, Raphidophyceae and Xantophyceae are grouped together in a highly supported clade, and this arrangement is consistent with the traditional phylogeny of such taxa, especially regarding Heterokonts [110]. We cannot undoubtedly assert that they belong to the same CHS family of land plants but surely they can be considered as type III PKS enzymes. The same assertion is valid for the homologs found in the haptophyte *Prymnesium parvum*, the dinoflagellate *Karlodinium micrum* and the oxyrrhidophyte *Oxyrrhis marina*. Such sequences are distantly related to those from other dinoflagellates or have homologs within the same species that are closer to other species in the phylogenetic tree (e.g., the case of *Oxyrrhis marina*).

In general, the finding of such homologs in all the aforementioned taxa is interesting since so far type III PKS genes from marine organisms were only known in some brown algae [111,112,113], dichtyochophytes [85] and ochrophytes [84]. Under the light of such findings, we support the current evolutionary scenario according to which type III PKS genes were acquired by an ancient lateral gene transfer event (likely from a bacterium) before the divergence of brown algae with pelagophytes and dichtyochophytes [84]. Our data also provide evidence for the fact that despite type III PKS homologs are absent in the genome, such as the diatoms *Phaeodactylum tricornutum* and *Thalassiosira pseudonana* [84], they occur in the transcriptomes of the congeneric (*T. minuscola* and *T. weissflogii*) or other (*Fragilariopsis kerguelensis*) diatom species. Furthermore, we provide evidence for the first time of the occurrence of type III PKS homologs in green algae. Indeed, we found three CHS/STS homologs within the transcriptome of the prasinophyte *Pyramimonas parkeae*, which were confirmed by domain analyses. Such preliminary results open up new scenarios about the evolution and the metabolic and ecological role of such enzymes in the taxa here investigated.

Since chalcone and stilbene synthases catalyse the sequential decarboxylative addition of three acetate units from malonyl-CoA to a p-coumaryl-CoA starter molecule [20], we also looked for the occurrence of the 4-Coumarate: CoA ligase (4CL), the enzyme responsible for the production of p-coumaryl-CoA. Our results indicate that homologs annotated as 4CL occurred in a limited number of taxa as Bacillariophyta, Dinophyceae, Pavlovophyceae and Raphidophyceae. Since we analysed transcriptomic data, the absence of 4CL homologs (or CHS/STS for the case above) in some taxa does not necessarily mean that such genes are absent in those organisms but simply that they were not expressed at the time of sampling. This constitutes a clear limitation of transcriptome over genome mining when searching for genes/pathways of interest. Nonetheless, to date, transcriptome mining remains a valuable resource if considering the disproportionate amount of microalgal transcriptomes over genomes available. Our knowledge of occurrence of 4CLs is limited to land plants and some streptophyte algae [86] and little is known about other photosynthetic lineages. In these taxa, this enzyme seems to be involved in the production of lignin-like compounds and defense mechanisms. However, many lands plants also possess several 4CL-like enzymes that are not involved in flavonoid or lignin biosynthesis but whose function is still unknown [114,115,116].

In our analysis, we found only two organisms that expressed both CHS/STS and 4CL-like enzymes, the raphidophyte *Heterosigma akashiwo* (strains CCMP3107 and NB), and the dinoflagellate *Kryptoperidinium foliaceum*. These microorganisms are phylogenetically distant, there is not much information regarding their bioactivities or biosynthetic pathways, and are hence the taxa of election for further investigations. These results give new insights into the presence of molecular machineries for the production of naringenin chalcone or resveratrol, or, at least, what their homologs do in land plants. Marine microalgae possessing type I and II PKS enzymes are already known to produce polyketides with applications in human health and biotechnology [117,118,119,120]. We demonstrated that several lineages of microalgae possess type III PKS resembling CHS/STS genes, which posed new questions on their possible functions in microalgae. From a biotechnological point of view, this discovery shed light on new biosynthetic pathways to be considered for the production of bioactive compounds from microalgae.

## Supplementary Materials

The following are available online at https://www.mdpi.com/2079-7737/9/5/110/s1, File S1: CHS/STS annotated homologs in fasta format, File S2: Final alignment of CHS/STS annotated homologs used for phylogenetic inference, File S3: 4CL annotated homologs in fasta format, File S4: Final alignment of 4CL annotated homologs used for phylogenetic inference, Table S1: List of MMETSP transcriptomes screened in the present study, with taxonomic information of taxa investigated, Table S2: List of ingroup and outgroup taxa utilised in CHS/STS (a) and 4CL (b) phylogenies.

## References

[1] Ingebrigtsen R.A., Hansen E., Andersen J.H., Eilertsen H.C. (2016). Light and temperature effects on bioactivity in diatoms. J. Appl. Phycol..

[2] Lauritano C., Ianora A. (2016). Marine Organisms with Anti-Diabetes Properties. Mar. Drugs.

[3] Lauritano C., Andersen J.H., Hansen E., Albrigtsen M., Escalera L., Esposito F., Helland K., Hanssen K.Ø., Romano G., Ianora A. (2016). Bioactivity Screening of Microalgae for Antioxidant, Anti-Inflammatory, Anticancer, Anti-Diabetes, and Antibacterial Activities. Front. Mar. Sci..

[4] Romano G., Costantini M., Sansone C., Lauritano C., Ruocco N., Ianora A. (2017). Marine microorganisms as a promising and sustainable source of bioactive molecules. Mar. Environ. Res..

[5] Brillatz T., Lauritano C., Jacmin M., Khamma S., Marcourt L., Righi D., Romano G., Esposito F., Ianora A., Queiroz E.F. (2018). Zebrafish-based identification of the antiseizure nucleoside inosine from the marine diatom *Skeletonema marinoi*. PLoS ONE.

[6] Giordano D., Costantini M., Coppola D., Lauritano C., Núñez Pons L., Ruocco N., di Prisco G., Ianora A., Verde C. (2018). Biotechnological Applications of Bioactive Peptides From Marine Sources. Advances in Microbial Physiology.

[7] Martínez Andrade K., Lauritano C., Romano G., Ianora A. (2018). Marine Microalgae with Anti-Cancer Properties. Mar. Drugs.

[8] Martínez K.A., Lauritano C., Druka D., Romano G., Grohmann T., Jaspars M., Martín J., Díaz C., Cautain B., de la Cruz M. (2019). Amphidinol 22, a New Cytotoxic and Antifungal Amphidinol from the Dinoflagellate *Amphidinium carterae*. Mar. Drugs.

[9] Lauritano C., Helland K., Riccio G., Andersen J.H., Ianora A., Hansen E.H. (2020). Lysophosphatidylcholines and Chlorophyll-Derived Molecules from the Diatom *Cylindrotheca closterium* with Anti-Inflammatory Activity. Mar. Drugs.

[10] Riccio G., Lauritano C. (2019). Microalgae with Immunomodulatory Activities. Mar. Drugs.

[11] Lauritano C., De Luca D., Ferrarini A., Avanzato C., Minio A., Esposito F., Ianora A. (2017). De novo transcriptome of the cosmopolitan dinoflagellate *Amphidinium carterae* to identify enzymes with biotechnological potential. Sci. Rep..

[12] Di Dato V., Di Costanzo F., Barbarinaldi R., Perna A., Ianora A., Romano G. (2019). Unveiling the presence of biosynthetic pathways for bioactive compounds in the *Thalassiosira rotula* transcriptome. Sci. Rep..

[13] Lauritano C., De Luca D., Amoroso M., Benfatto S., Maestri S., Racioppi C., Esposito F., Ianora A. (2019). New molecular insights on the response of the green alga *Tetraselmis suecica* to nitrogen starvation. Sci. Rep..

[14] Elagoz A.M., Ambrosino L., Chiara L. (2020). De novo transcriptome of the diatom *Cylindrotheca closterium* identifies genes involved in the metabolism of anti-inflammatory compounds. Sci. Rep..

[15] Austin M.B., Izumikawa M., Bowman M.E., Udwary D.W., Ferrer J.L., Moore B.S., Noel J.P. (2004). Crystal structure of a bacterial type III polyketide synthase and enzymatic control of reactive polyketide intermediates. J. Biol. Chem..

[16] Austin M.B., Noel J.P. (2003). The chalcone synthase superfamily of type III polyketide synthases. Nat. Prod. Rep..

[17] Funa N., Awakawa T., Horinouchi S. (2007). Pentaketide resorcylic acid synthesis by type III polyketide synthase from *Neurospora crassa*. J. Biol. Chem..

[18] Seshime Y., Juvvadi P.R., Fujii I., Kitamoto K. (2005). Discovery of a novel superfamily of type III polyketide synthases in *Aspergillus oryzae*. Biochem. Biophys. Res. Commun..

[19] Abe I., Morita H. (2010). Structure and function of the chalcone synthase superfamily of plant type III polyketide synthases. Nat. Prod. Rep..

[20] Pfeifer B.A., Khosla C. (2001). Biosynthesis of Polyketides in Heterologous Hosts. Microbiol. Mol. Biol. Rev..

[21] Flores-Sanchez I.J., Verpoorte R. (2009). Plant Polyketide Synthases: A fascinating group of enzymes. Plant Physiol. Biochem..

[22] Schröder J., Schröder G. (1990). Stilbene and Chalcone Synthases: Related Enzymes with Key Functions in Plant-Specific Pathways. Z. Naturforsch. Sect. C J. Biosci..

[23] Goodwin P.H., Hsiang T., Erickson L. (2000). A comparison of stilbene and chalcone synthases including a new stilbene synthase gene from *Vitis riparia* cv. Gloire de Montpellier. Plant Sci..

[24] Tropf S., Lanz T., Rensing S.A., Schröder J., Schröder G. (1994). Evidence that stilbene synthases have developed from chalcone synthases several times in the course of evolution. J. Mol. Evol..

[25] Rao V.P., Kiran S. (2017). Flavonoid: A review on Naringenin. J. Pharmacogn. Phytochem..

[26] Patel K., Singh G.K., Patel D.K. (2018). A Review on Pharmacological and Analytical Aspects of Naringenin. Chin. J. Integr. Med..

[27] Lewis C.E., Walker J.R.L., Lancaster J.E., Sutton K.H. (1998). Determination of anthocyanins, flavonoids and phenolic acids in potatoes. I: Coloured cultivars of *Solanum tuberosum* L.. J. Sci. Food Agric..

[28] Tomás-Barberán F.A., Clifford M.N. (2000). Flavanones, chalcones and dihydrochalcones—Nature, occurrence and dietary burden. J. Sci. Food Agric..

[29] Langcake P., Pryce R.J. (1976). The production of resveratrol by *Vitis vinifera* and other members of the Vitaceae as a response to infection or injury. Physiol. Plant Pathol..

[30] Sobolev V.S., Cole R.J. (1999). *trans*-Resveratrol Content in Commercial Peanuts and Peanut Products. J. Agric. Food Chem..

[31] Kodan A., Kuroda H., Sakai F. (2002). A stilbene synthase from Japanese red pine (*Pinus densiflora*): Implications for phytoalexin accumulation and down-regulation of flavonoid biosynthesis. Proc. Natl. Acad. Sci. USA.

[32] Lyons M.M., Yu C., Toma R.B., Cho S.Y., Reiboldt W., Lee J., van Breemen R.B. (2003). Resveratrol in Raw and Baked Blueberries and Bilberries. J. Agric. Food Chem..

[33] Wibowo A., Ahmat N., Hamzah A.S., Ismail N.H., Ahmad R., Jaafar F.M. (2012). Resveratrol oligomers from the stem bark of Dryobalanops aromatica. Biochem. Syst. Ecol..

[34] Chen H., Tuck T., Ji X., Zhou X., Kelly G., Cuerrier A., Zhang J. (2013). Quality Assessment of Japanese Knotweed (*Fallopia japonica*) Grown on Prince Edward Island as a Source of Resveratrol. J. Agric. Food Chem..

[35] Jeandet P., Bessis R., Gautheron B. (1991). The Production of Resveratrol (3,5,4’-trihydroxystilbene) by Grape Berries in Different Developmental Stages. Am. J. Enol. Vitic..

[36] Siemann E.H., Creasy L.L. (1992). Concentration of the Phytoalexin Resveratrol in Wine. Am. J. Enol. Vitic..

[37] Romero-Pérez A.I., Ibern-Gómez M., Lamuela-Raventós R.M., de la Torre-Boronat M.C. (1999). Piceid, the Major Resveratrol Derivative in Grape Juices. J. Agric. Food Chem..

[38] Hahlbrock K., Scheel D. (1989). Physiology and Molecular Biology of Phenylpropanoid Metabolism. Annu. Rev. Plant Physiol. Plant Mol. Biol..

[39] Akada S., Kung S.D., Dube S.K. (1993). Nucleotide sequence of a soybean chalcone synthase gene with a possible role in ultraviolet-B sensitivity, Gmchs6. Plant Physiol..

[40] Dao T.T.H., Linthorst H.J.M., Verpoorte R. (2011). Chalcone synthase and its functions in plant resistance. Phytochem. Rev..

[41] Walle T. (2007). Methoxylated flavones, a superior cancer chemopreventive flavonoid subclass?. Semin. Cancer Biol..

[42] Meiyanto E., Hermawan A. (2012). Anindyajati Natural products for cancer-targeted therapy: Citrus flavonoids as potent chemopreventive agents. Asian Pac. J. Cancer Prev..

[43] Raffa D., Maggio B., Raimondi M.V., Plescia F., Daidone G. (2017). Recent discoveries of anticancer flavonoids. Eur. J. Med. Chem..

[44] Pietta P.-G. (2000). Flavonoids as Antioxidants. J. Nat. Prod..

[45] Nicole Cotelle B.S.P. (2005). Role of Flavonoids in Oxidative Stress. Curr. Top. Med. Chem..

[46] Procházková D., Boušová I., Wilhelmová N. (2011). Antioxidant and prooxidant properties of flavonoids. Fitoterapia.

[47] Zwaagstra M.E., Timmerman H., Tamura M., Tohma T., Wada Y., Onogi K., Zhang M.-Q. (1997). Synthesis and Structure—Activity Relationships of Carboxylated Chalcones: A Novel Series of *CysLT*_1_ (LTD_4_) Receptor Antagonists. J. Med. Chem..

[48] Tanaka T., Takahashi R. (2013). Flavonoids and Asthma. Nutrients.

[49] Flores-Flores A., Estrada-Soto S., Millán-Pacheco C., Bazán-Perkins B., Villalobos-Molina R., Moreno-Fierros L., Hernández-Pando R., García-Jiménez S., Rivera-Leyva J.C. (2019). Functional mechanism of tracheal relaxation, antiasthmatic, and toxicological studies of 6-hydroxyflavone. Drug Dev. Res..

[50] Kim H.P., Son K.H., Chang H.W., Kang S.S. (2004). Anti-inflammatory Plant Flavonoids and Cellular Action Mechanisms. J. Pharmacol. Sci..

[51] Pan M.H., Lai C.S., Ho C.T. (2010). Anti-inflammatory activity of natural dietary flavonoids. Food Funct..

[52] Serafini M., Peluso I., Raguzzini A. (2010). Flavonoids as anti-inflammatory agents. Proc. Nutr. Soc..

[53] Takahashi T., Kokubo R., Sakaino M. (2004). Antimicrobial activities of eucalyptus leaf extracts and flavonoids from *Eucalyptus maculata*. Lett. Appl. Microbiol..

[54] Cushnie T.P.T., Lamb A.J. (2005). Antimicrobial activity of flavonoids. Int. J. Antimicrob. Agents.

[55] Sarbu L.G., Bahrin L.G., Babii C., Stefan M., Birsa M.L. (2019). Synthetic flavonoids with antimicrobial activity: A review. J. Appl. Microbiol..

[56] Khaomek P., Ichino C., Ishiyama A., Sekiguchi H., Namatame M., Ruangrungsi N., Saifah E., Kiyohara H., Otoguro K., Omura S. (2008). In vitro antimalarial activity of prenylated flavonoids from *Erythrina fusca*. J. Nat. Med..

[57] Bero J., Frédérich M., Quetin-Leclercq J. (2009). Antimalarial compounds isolated from plants used in traditional medicine. J. Pharm. Pharmacol..

[58] Pan W.-H., Xu X.-Y., Shi N., Tsang S., Zhang H.-J. (2018). Antimalarial Activity of Plant Metabolites. Int. J. Mol. Sci..

[59] Cao Y., Fu Z.-D., Wang F., Liu H.Y., Han R. (2005). Anti-angiogenic activity of resveratrol, a natural compound from medicinal plants. J. Asian Nat. Prod. Res..

[60] Trapp V., Parmakhtiar B., Papazian V., Willmott L., Fruehauf J.P. (2010). Anti-angiogenic effects of resveratrol mediated by decreased VEGF and increased TSP1 expression in melanoma-endothelial cell co-culture. Angiogenesis.

[61] Kasiotis K.M., Pratsinis H., Kletsas D., Haroutounian S.A. (2013). Resveratrol and related stilbenes: Their anti-aging and anti-angiogenic properties. Food Chem. Toxicol..

[62] Sharma S., Misra C.S., Arumugam S., Roy S., Shah V., Davis J.A., Shirumalla R.K., Ray A. (2011). Antidiabetic activity of resveratrol, a known SIRT1 activator in a genetic model for type-2 diabetes. Phyther. Res..

[63] Szkudelski T., Szkudelska K. (2011). Anti-diabetic effects of resveratrol. Ann. NY. Acad. Sci..

[64] Oyenihi O.R., Oyenihi A.B., Adeyanju A.A., Oguntibeju O.O. (2016). Antidiabetic Effects of Resveratrol: The Way Forward in Its Clinical Utility. J. Diabetes Res..

[65] Chen Y., Tseng S.H., Lai H.S., Chen W.J. (2004). Resveratrol-induced cellular apoptosis and cell cycle arrest in neuroblastoma cells and antitumor effects on neuroblastoma in mice. Surgery.

[66] Harikumar K.B., Kunnumakkara A.B., Sethi G., Diagaradjane P., Anand P., Pandey M.K., Gelovani J., Krishnan S., Guha S., Aggarwal B.B. (2010). Resveratrol, a multitargeted agent, can enhance antitumor activity of gemcitabine in vitro and in orthotopic mouse model of human pancreatic cancer. Int. J. Cancer.

[67] Gao F., Deng G., Liu W., Zhou K., Li M. (2017). Resveratrol suppresses human hepatocellular carcinoma via targeting HGF-c-Met signaling pathway. Oncol. Rep..

[68] Campagna M., Rivas C. (2010). Antiviral activity of resveratrol. Biochem. Soc. Trans..

[69] Abba Y., Hassim H., Hamzah H., Noordin M.M. (2015). Antiviral Activity of Resveratrol against Human and Animal Viruses. Adv. Virol..

[70] Mohd A., Zainal N., Tan K.-K., AbuBakar S. (2019). Resveratrol affects Zika virus replication in vitro. Sci. Rep..

[71] Hung L. (2000). Cardioprotective effect of resveratrol, a natural antioxidant derived from grapes. Cardiovasc. Res..

[72] Riba A., Deres L., Sumegi B., Toth K., Szabados E., Halmosi R. (2017). Cardioprotective Effect of Resveratrol in a Postinfarction Heart Failure Model. Oxidative Med. Cell. Longev..

[73] Abdelgawad I., Grant M., Zordoky B. (2019). Leveraging the Cardio-Protective and Anticancer Properties of Resveratrol in Cardio-Oncology. Nutrients.

[74] Albani D., Polito L., Signorini A., Forloni G. (2010). Neuroprotective properties of resveratrol in different neurodegenerative disorders. BioFactors.

[75] Tang B.L. (2010). Resveratrol is neuroprotective because it is not a direct activator of Sirt1-A hypothesis. Brain Res. Bull..

[76] Bastianetto S., Ménard C., Quirion R. (2015). Neuroprotective action of resveratrol. Biochim. Biophys. Acta Mol. Basis Dis..

[77] Soleas G.J., Diamandis E.P., Goldberg D.M. (2001). The world of resveratrol. Adv. Exp. Med. Biol..

[78] Khanduja K.L., Bhardwaj A. (2003). Stable free radical scavenging and antiperoxidative properties of resveratrol compared in vitro with some other bioflavonoids. Indian J. Biochem. Biophys..

[79] Protić D., Beleslin-Čokić B., Spremović-Rađenović S., Radunović N., Heinle H., Šćepanović R., Gojković Bukarica L. (2014). The Different Effects of Resveratrol and Naringenin on Isolated Human Umbilical Vein: The Role of ATP-Sensitive K^+^ Channels. Phyther. Res..

[80] Jiang C., Kim S.Y., Suh D.Y. (2008). Divergent evolution of the thiolase superfamily and chalcone synthase family. Mol. Phylogenetics Evol..

[81] Austin M.B., Bowman M.E., Ferrer J.L., Schröder J., Noel J.P. (2004). An aldol switch discovered in stilbene synthases mediates cyclization specificity of type III polyketide synthases. Chem. Biol..

[82] Yamazaki Y., Suh D.Y., Sitthithaworn W., Ishiguro K., Kobayashi Y., Shibuya M., Ebizuka Y., Sankawa U. (2001). Diverse chalcone synthase superfamily enzymes from the most primitive vascular plant, *Psilotum nudum*. Planta.

[83] Harashima S., Takano H., Ono K., Takio S. (2004). Chalcone synthase-like gene in the liverwort, *Marchantia paleacea* var. diptera. Plant Cell Rep..

[84] Meslet-Cladière L., Delage L., Leroux C.J.J., Goulitquer S., Leblanc C., Creis E., Gall E.A., Stiger-Pouvreau V., Czjzek M., Potin P. (2013). Structure/function analysis of a type III polyketide synthase in the brown alga *Ectocarpus siliculosus* reveals a biochemical pathway in phlorotannin monomer biosynthesis. Plant Cell.

[85] Dittami S., Riisberg I., John U., Orr R.J., Jakobsen K.S., Edvardsen B. (2012). Analysis of expressed sequence tags from the marine microalga *Pseudochattonella farcimen* (Dictyochophyceae). Protist.

[86] De Vries J., De Vries S., Slamovits C.H., Rose L.E., Archibald J.M. (2017). How embryophytic is the biosynthesis of phenylpropanoids and their derivatives in streptophyte algae?. Plant Cell Physiol..

[87] Wisecaver J.H., Hackett J.D. (2011). Dinoflagellate Genome Evolution. Annu. Rev. Microbiol..

[88] Casabianca S., Cornetti L., Capellacci S., Vernesi C., Penna A. (2017). Genome complexity of harmful microalgae. Harmful Algae.

[89] Guarnieri M.T., Nag A., Smolinski S.L., Darzins A., Seibert M., Pienkos P.T. (2011). Examination of triacylglycerol biosynthetic pathways via de novo transcriptomic and proteomic analyses in an unsequenced microalga. PLoS ONE.

[90] Bochenek M., Etherington G.J., Koprivova A., Mugford S.T., Bell T.G., Malin G., Kopriva S. (2013). Transcriptome analysis of the sulfate deficiency response in the marine microalga *Emiliania huxleyi*. New Phytol..

[91] Li Q., Liu J., Zhang L., Liu Q. (2014). De novo transcriptome analysis of an aerial microalga *Trentepohlia jolithus*: Pathway description and gene discovery for carbon fixation and carotenoid biosynthesis. PLoS ONE.

[92] Ashworth J., Ralph P.J. (2018). An explorable public transcriptomics compendium for eukaryotic microalgae. BioRxiv.

[93] Keeling P.J., Burki F., Wilcox H.M., Allam B., Allen E.E., Amaral-Zettler L.A., Armbrust E.V., Archibald J.M., Bharti A.K., Bell C.J. (2014). The Marine Microbial Eukaryote Transcriptome Sequencing Project (MMETSP): Illuminating the Functional Diversity of Eukaryotic Life in the Oceans through Transcriptome Sequencing. PLoS Biol..

[94] Altschul S.F., Gish W., Miller W., Myers E.W., Lipman D.J. (1990). Basic local alignment search tool. J. Mol. Biol..

[95] Huerta-Cepas J., Forslund K., Coelho L.P., Szklarczyk D., Jensen L.J., von Mering C., Bork P. (2017). Fast Genome-Wide Functional Annotation through Orthology Assignment by eggNOG-Mapper. Mol. Biol. Evol..

[96] Huerta-Cepas J., Szklarczyk D., Heller D., Hernández-Plaza A., Forslund S.K., Cook H., Mende D.R., Letunic I., Rattei T., Jensen L.J. (2019). eggNOG 5.0: A hierarchical, functionally and phylogenetically annotated orthology resource based on 5090 organisms and 2502 viruses. Nucleic Acids Res..

[97] Cukovic D., Ehlting J., VanZiffle J.A., Douglas C.J. (2001). Structure and evolution of 4-coumarate: Coenzyme A ligase (4CL) gene families. Biol. Chem..

[98] Papadopoulos J.S., Agarwala R. (2007). COBALT: Constraint-based alignment tool for multiple protein sequences. Bioinformatics.

[99] Capella-Gutiérrez S., Silla-Martínez J.M., Gabaldón T. (2009). trimAl: A tool for automated alignment trimming in large-scale phylogenetic analyses. Bioinformatics.

[100] Guindon S., Gascuel O. (2003). A Simple, Fast, and Accurate Algorithm to Estimate Large Phylogenies by Maximum Likelihood. Syst. Biol..

[101] Lefort V., Longueville J.-E., Gascuel O. (2017). SMS: Smart Model Selection in PhyML.. Mol. Biol. Evol..

[102] Anisimova M., Gascuel O. (2006). Approximate Likelihood-Ratio Test for Branches: A Fast, Accurate, and Powerful Alternative. Syst. Biol..

[103] Jones P., Binns D., Chang H.-Y., Fraser M., Li W., McAnulla C., McWilliam H., Maslen J., Mitchell A., Nuka G. (2014). InterProScan 5: Genome-scale protein function classification. Bioinformatics.

[104] Sagar M., Pandey N., Qamar N., Singh B., Shukla A. (2015). Domain analysis of 3 Keto Acyl-CoA synthase for structural variations in *Vitis vinifera* and *Oryza brachyantha* using comparative modelling. Interdiscip. Sci. Comput. Life Sci..

[105] Le S.Q., Gascuel O. (2008). An Improved General Amino Acid Replacement Matrix. Mol. Biol. Evol..

[106] Pawlowiez R., Morey J.S., Darius H.T., Chinain M., Van Dolah F.M. (2014). Transcriptome sequencing reveals single domain Type I-like polyketide synthases in the toxic dinoflagellate *Gambierdiscus polynesiensis*. Harmful Algae.

[107] Meyer J.M., Rödelsperger C., Eichholz K., Tillmann U., Cembella A., McGaughran A., John U. (2015). Transcriptomic characterisation and genomic glimps into the toxigenic dinoflagellate *Azadinium spinosum*, with emphasis on polykeitde synthase genes. BMC Genom..

[108] Kohli G.S., John U., Figueroa R.I., Rhodes L.L., Harwood D.T., Groth M., Bolch C.J.S., Murray S.A. (2015). Polyketide synthesis genes associated with toxin production in two species of *Gambierdiscus* (Dinophyceae). BMC Genom..

[109] Kohli G.S., John U., Van Dolah F.M., Murray S.A. (2016). Evolutionary distinctiveness of fatty acid and polyketide synthesis in eukaryotes. ISME J..

[110] Riisberg I., Orr R.J.S., Kluge R., Shalchian-Tabrizi K., Bowers H.A., Patil V., Edvardsen B., Jakobsen K.S. (2009). Seven Gene Phylogeny of Heterokonts. Protist.

[111] Wong T.K.M., Ho C.L., Lee W.W., Rahim R.A., Phang S.M. (2007). Analyses of expressed sequence tags from *Sargassum binderi* (Phaeophyta). J. Phycol..

[112] Pearson G.A., Hoarau G., Lago-Leston A., Coyer J.A., Kube M., Reinhardt R., Henckel K., Serrão E.T.A., Corre E., Olsen J.L. (2010). An expressed sequence tag analysis of the intertidal brown seaweeds *Fucus serratus* (L.) and *F. vesiculosus* (L.) (Heterokontophyta, Phaeophyceae) in response to abiotic stressors. Mar. Biotechnol..

[113] Baharum H., Morita H., Tomitsuka A., Lee F.C., Ng K.Y., Rahim R.A., Abe I., Ho C.L. (2011). Molecular Cloning, Modeling, and Site-Directed Mutagenesis of Type III Polyketide Synthase from *Sargassum binderi* (Phaeophyta). Mar. Biotechnol..

[114] Raes J., Rohde A., Christensen J.H., Van De Peer Y., Boerjan W. (2003). Genome-Wide Characterization of the Lignification Toolbox in *Arabidopsis*. Plant Physiol..

[115] Ehlting J., Mattheus N., Aeschliman D.S., Li E., Hamberger B., Cullis I.F., Zhuang J., Kaneda M., Mansfield S.D., Samuels L. (2005). Global transcript profiling of primary stems from *Arabidopsis thaliana* identifies candidate genes for missing links in lignin biosynthesis and transcriptional regulators of fiber differentiation. Plant J..

[116] Cao Y., Han Y., Li D., Lin Y., Cai Y. (2016). Systematic Analysis of the 4-Coumarate: Coenzyme A Ligase (4CL) Related Genes and Expression Profiling during Fruit Development in the Chinese Pear. Genes.

[117] Fenical W., Jensen P.R., Kauffman C., Mayhead S.L., Faulkner D.J., Sincich C., Rao M.R., Kantorowski E.J., West L.M., Strangman W.K. (2003). New anticancer drugs from cultured and collected marine organisms. Pharm. Biol..

[118] Bao J., Sun Y.L., Zhang X.Y., Han Z., Gao H.C., He F., Qian P.Y., Qi S.H. (2013). Antifouling and antibacterial polyketides from marine gorgonian coral-associated fungus *Penicillium* sp. SCSGAF 0023. J. Antibiot..

[119] Kobayashi J., Kubota T. (2007). Bioactive macrolides and polyketides from marine dinoflagellates of the genus *Amphidinium*. J. Nat. Prod..

[120] Kumagai K., Minamida M., Akakabe M., Tsuda M., Konishi Y., Tominaga A., Tsuda M., Fukushi E., Kawabata J. (2015). Amphirionin-2, a novel linear polyketide with potent cytotoxic activity from a marine dinoflagellate *Amphidinium* species. Bioorg. Med. Chem. Lett..

