# Supplementary material for: In Silico Identification of Type III PKS Chalcone and Stilbene Synthase Homologs in Marine Photosynthetic Organisms
Source: Biology (Basel). 2020 May 22;9(5):110. doi: 10.3390/biology9050110 (PMC7284882; doi:10.3390/biology9050110)
Supplement: Supplementary file 1 [file biology-09-00110-s001.zip › Supplementary/supplementary Tables-final.docx]

**Table S1.** List of MMETSP transcriptomes screened in the present study, with taxonomic information of taxa investigated.

|  |  |  | **CHS/STS** | | **4CL** | |
| --- | --- | --- | --- | --- | --- | --- |
| **Transcriptomes analysed** | **Phylum** | **Class** | **Retrieved homologs** | **Annotated homologs** | **Retrieved homologs** | **Annotated homologs** |
| *Acanthoeca*-like sp. | Choanozoa | Choanoflagellatea |  |  |  |  |
| *Alexandrium fundyense* CCMP1719 | Miozoa | Dinophyceae |  |  |  |  |
| *Alexandrium monilatum* CCMP3105 | Miozoa | Dinophyceae |  |  |  |  |
| *Alexandrium temarense* CCMP1771 | Miozoa | Dinophyceae |  |  |  |  |
| *Amphidinium carterae* CCMP1314 | Miozoa | Dinophyceae |  |  |  |  |
| *Amphiprora* sp. | Bacillariophyta | Bacillariophyceae |  |  |  |  |
| *Amphora coffeaeformis* CCMP127 | Bacillariophyta | Bacillariophyceae |  |  |  |  |
| *Aplanochytrium* sp. PBS07 | Bigyra | Labyrinthulea |  |  |  |  |
| *Aplanochytrium stocchinoi* GSBS06 | Bigyra | Labyrinthulea |  |  |  |  |
| *Asterionellopsis glacialis* CCMP134 | Bacillariophyta | Bacillariophyceae |  |  |  |  |
| *Aurantiochytrium limacinum* ATCCMYA1381 | Bigyra | Labyrinthulea |  |  |  |  |
| *Aureococcus anophagefferens* CCMP1850 | Ochrophyta | Pelagophyceae |  |  |  |  |
| *Aureoumbra lagunensis* CCMP1510 | Ochrophyta | Pelagophyceae |  |  |  |  |
| *Azadinium spinosum* 3D9 | Miozoa | Dinophyceae |  |  |  |  |
| *Ceratium fusus* PA161109 | Miozoa | Dinophyceae |  |  |  |  |
| *Chaetoceros affinis* CCMP159 | Bacillariophyta | Bacillariophyceae |  |  |  |  |
| *Chaetoceros curvisetus* | Bacillariophyta | Bacillariophyceae |  |  |  |  |
| *Chaetoceros debilis* MM31A 1 | Bacillariophyta | Bacillariophyceae |  |  |  |  |
| *Chaetoceros neogracile* CCMP1317 | Bacillariophyta | Bacillariophyceae |  |  |  |  |
| *Chattonella subsalsa* CCMP2191 | Ochrophyta | Raphidophyceae |  |  |  |  |
| *Chrysochromulina polylepis* CCMP1757 | Haptophyta | Coccolithophyceae |  |  |  |  |
| *Corethron pennatum* L29A3 | Bacillariophyta | Coscinodiscophyceae |  |  |  |  |
| *Crypthecodinium cohnii* Seligo | Miozoa | Dinophyceae |  |  |  |  |
| *Debaryomyces hansenii* J26 | Ascomycota | Saccharomycetes |  |  |  |  |
| *Dinobryon* sp. UTEXLB2267 | Ochrophyta | Chrysophyceae |  |  |  |  |
| *Ditylum brightwellii* GSO103 | Bacillariophyta | Mediophyceae |  |  |  |  |
| *Ditylum brightwellii* GSO104 | Bacillariophyta | Mediophyceae |  |  |  |  |
| *Ditylum brightwellii* GSO105 | Bacillariophyta | Mediophyceae |  |  |  |  |
| *Dunaliella tertiolecta* CCMP1320 | Chlorophyta | Chlorophyceae |  |  |  |  |
| *Durinskia baltica* CSIRO CS 38 | Miozoa | Dinophyceae |  |  |  |  |
| *Emiliania huxleyi* 374 | Haptophyta | Coccolithophyceae |  |  |  |  |
| *Emiliania huxleyi* 379 | Haptophyta | Coccolithophyceae |  |  |  |  |
| *Emiliania huxleyi* CCMP370 | Haptophyta | Coccolithophyceae |  |  |  |  |
| *Emiliania huxleyi* PLYM219 | Haptophyta | Coccolithophyceae |  |  |  |  |
| *Euplotes focardii* TN1 | Ciliophora | Spirotrichea |  |  |  |  |
| *Eutreptiella gymnastica-*like CCMP1594 | Euglenozoa | Euglenophyceae |  |  |  |  |
| *Extubocellulus spinifer* CCMP396 | Bacillariophyta | Mediophyceae |  |  |  |  |
| *Favella taraikaensis* FeNarragansettBay | Ciliophora | Oligotrichea |  |  |  |  |
| *Fragilariopsis kerguelensis* L26 C5 | Bacillariophyta | Bacillariophyceae |  |  |  |  |
| *Fragilariopsis kerguelensis* L2 C3 | Bacillariophyta | Bacillariophyceae |  |  |  |  |
| *Gephyrocapsa oceanica* RCC1303 | Haptophyta | Coccolithophyceae |  |  |  |  |
| *Glenodinium foliaceum* CCAP1116 3 | Miozoa | Dinophyceae |  |  |  |  |
| *Gloeochaete witrockiana* SAG46 84 | Glaucophyta | Glaucophyceae |  |  |  |  |
| *Goniomonas pacifica* CCMP1869 | Cryptophyta | Cryptophyceae |  |  |  |  |
| *Heterosigma akashiwo* CCMP2393 | Ochrophyta | Raphidophyceae |  |  |  |  |
| *Heterosigma akashiwo* CCMP3107 | Ochrophyta | Raphidophyceae |  |  |  |  |
| *Heterosigma akashiwo* CCMP452 | Ochrophyta | Raphidophyceae |  |  |  |  |
| *Heterosigma akashiwo* NB | Ochrophyta | Raphidophyceae |  |  |  |  |
| *Isochrysis galbana* CCMP1323 | Haptophyta | Coccolithophyceae |  |  |  |  |
| *Isochrysis* sp. CCMP1244 | Haptophyta | Coccolithophyceae |  |  |  |  |
| *Isochrysis* sp. CCMP1324 | Haptophyta | Coccolithophyceae |  |  |  |  |
| *Karenia brevis* CCMP2229 | Miozoa | Dinophyceae |  |  |  |  |
| *Karenia brevis* SP1 | Miozoa | Dinophyceae |  |  |  |  |
| *Karenia brevis* SP3 | Miozoa | Dinophyceae |  |  |  |  |
| *Karenia brevis* Wilson | Miozoa | Dinophyceae |  |  |  |  |
| *Karlodinium micrum* CCMP2283 | Miozoa | Dinophyceae |  |  |  |  |
| *Kryptoperidinium foliaceum* CCMP1326 | Miozoa | Dinophyceae |  |  |  |  |
| *Lingulodinium polyedra* CCMP1738 | Miozoa | Dinophyceae |  |  |  |  |
| *Lotharella globosa* CCCM811 | Cercozoa | Chlorarachniophyceae |  |  |  |  |
| *Micromonas* sp. CCMP2099 | Chlorophyta | Mamiellophyceae |  |  |  |  |
| *Micromonas* sp. NEPCC29 | Chlorophyta | Mamiellophyceae |  |  |  |  |
| *Micromonas* sp. RCC472 | Chlorophyta | Mamiellophyceae |  |  |  |  |
| *Nitzschia punctata* CCMP561 | Bacillariophyta | Bacillariophyceae |  |  |  |  |
| *Ochromonas* sp. CCMP1393 | Ochrophyta | Chrysophyceae |  |  |  |  |
| *Oxyrrhis marina* | Miozoa | Oxyrrhidophyceae |  |  |  |  |
| *Oxyrrhis marina* CCMP1795 | Miozoa | Oxyrrhidophyceae |  |  |  |  |
| *Oxyrrhis marina* LB1974 | Miozoa | Oxyrrhidophyceae |  |  |  |  |
| *Paraphysomonas imperforata* PA2 | Ochrophyta | Chrysophyceae |  |  |  |  |
| *Pavlova* sp. CCMP459 | Haptophyta | Pavlovophyceae |  |  |  |  |
| *Pelagococcus subviridis* CCMP1429 | Ochrophyta | Pelagophyceae |  |  |  |  |
| *Pelagomonas calceolata* CCMP1756 | Ochrophyta | Pelagophyceae |  |  |  |  |
| *Peridinium aciculiferum* PAER 2 | Miozoa | Dinophyceae |  |  |  |  |
| *Perkinsus chesapeaki* ATCC PRA 65 | Miozoa | Perkinsea |  |  |  |  |
| *Perkinsus marinus* ATCC50439 | Miozoa | Perkinsea |  |  |  |  |
| *Picocystis salinarum* CCMP1897 | Chlorophyta | Picocystophyceae |  |  |  |  |
| *Pleurochrysis carterae* CCMP645 | Haptophyta | Coccolithophyceae |  |  |  |  |
| *Proboscia alata* PI D3 | Bacillariophyta | Coscinodiscophyceae |  |  |  |  |
| *Prorocentrum minimum* CCMP1329 | Miozoa | Dinophyceae |  |  |  |  |
| *Prorocentrum minimum* CCMP2233 | Miozoa | Dinophyceae |  |  |  |  |
| *Prymnesium parvum* Texoma1 | Haptophyta | Coccolithophyceae |  |  |  |  |
| *Pseudo-nitzschia australis* 10249 10 AB | Bacillariophyta | Bacillariophyceae |  |  |  |  |
| *Pseudo-nitzschia fradulenta* WWA7 | Bacillariophyta | Bacillariophyceae |  |  |  |  |
| *Pseudopedinella elastica* CCMP716 | Ochrophyta | Dictyochophyceae |  |  |  |  |
| *Pteridomonas danica* PT | Ochrophyta | Dictyochophyceae |  |  |  |  |
| *Pyramimonas parkeae* CCMP726 | Chlorophyta | Pyramimonadophyceae |  |  |  |  |
| *Rhodella maculata* CCMP736 | Rhodophyta | Rhodellophyceae |  |  |  |  |
| *Rhodomonas* sp. CCMP768 | Cryptophyta | Cryptophyceae |  |  |  |  |
| *Schizochytrium aggregatum* ATCC28209 | Bigyra | Labyrinthulea |  |  |  |  |
| *Scrippsiella hangoei* SHTV5 | Miozoa | Dinophyceae |  |  |  |  |
| *Scrippsiella hangoei*-like SHHI 4 | Miozoa | Dinophyceae |  |  |  |  |
| *Scrippsiella trochoidea* CCMP3099 | Miozoa | Dinophyceae |  |  |  |  |
| *Skeletonema dohrnii* SkelB | Bacillariophyta | Mediophyceae |  |  |  |  |
| *Skeletonema marinoi* SkelA | Bacillariophyta | Mediophyceae |  |  |  |  |
| *Skeletonema menzelii* CCMP793 | Bacillariophyta | Mediophyceae |  |  |  |  |
| *Symbiodinium kawagutii* CCMP2468 | Miozoa | Dinophyceae |  |  |  |  |
| *Symbiodinium* sp. C1 | Miozoa | Dinophyceae |  |  |  |  |
| *Symbiodinium* sp. C15 | Miozoa | Dinophyceae |  |  |  |  |
| *Symbiodinium* sp. CCMP2430 | Miozoa | Dinophyceae |  |  |  |  |
| *Symbiodinium* sp. Mp | Miozoa | Dinophyceae |  |  |  |  |
| *Tetraselmis striata* LANL1001 | Chlorophyta | Chlorodendrophyceae |  |  |  |  |
| *Thalassionema nitzschioides* L26 B | Bacillariophyta | Bacillariophyceae |  |  |  |  |
| *Thalassiosira antarctica* CCMP982 | Bacillariophyta | Mediophyceae |  |  |  |  |
| *Thalassiosira gravida* GMp14c1 | Bacillariophyta | Mediophyceae |  |  |  |  |
| *Thalassiosira miniscula* CCMP1093 | Bacillariophyta | Mediophyceae |  |  |  |  |
| *Thalassiosira oceanica* CCMP1005 | Bacillariophyta | Mediophyceae |  |  |  |  |
| *Thalassiosira rotula* CCMP3096 | Bacillariophyta | Mediophyceae |  |  |  |  |
| *Thalassiosira rotula* GSO102 | Bacillariophyta | Mediophyceae |  |  |  |  |
| *Thalassiosira weissflogii* CCMP1010 | Bacillariophyta | Mediophyceae |  |  |  |  |
| *Thalassiosira weissflogii* CCMP1336 | Bacillariophyta | Mediophyceae |  |  |  |  |
| *Thalassiothrix antarctica* L6 D1 | Bacillariophyta | Bacillariophyceae |  |  |  |  |
| *Thraustochytrium* sp. LLF1b | Bigyra | Labyrinthulea |  |  |  |  |
| *Vaucheria litorea* CCMP2940 | Ochrophyta | Xanthophyceae |  |  |  |  |

Note: "annotated homologs" refers to functionally annotated homolog sequences. In red are indicated transcriptomes of non-photosynthetic organisms whose sequences have been used as internal controls in cases (4CL) where only distantly related outgroups were available. Green cells indicate presence of homologs in the transcriptomes.

**Table S2.** List of ingroup and outgroup taxa utilised in CHS/STS (a) and 4CL (b) phylogenies.

**(a)**

| **Ingroup Taxa** | **Accession Number** |
| --- | --- |
| *Arachis hypogaea* (STS) | CAA00091 |
| *Betula pendula* (CHS) | CAA71904 |
| *Brassica napus* (CHS) | XP013677202 |
| *Bromheadia finlaysoniana* (CHS) | AAB62876 |
| *Callistephus chinensis* (CHS) | CAA91930 |
| *Chrysosplenium americanum* (CHS) | AAB54075 |
| *Glycine max* (CHS) | P24826 |
| *Ipomoea nil* (CHS) | BAA21788 |
| *Juglans nigra* x *J. regia* (CHS) | CAA64452 |
| *Oryza sativa* Japonica Group (CHS) | BAA19186 |
| *Perilla frutescens* (CHS) | BAA19656 |
| *Pinus strobus* (CHS) | CAA06077 |
| *Pinus strobus* (STS) | CAA87012 |
| *Pinus sylvestris* (CHS) | CAA43166 |
| *Psilotum nudum* (CHS) | BAA87922 |
| *Psilotum nudum* (STS) | BAA87925 |
| *Raphanus sativus* (CHS) | AAB87072 |
| *Scutellaria baicalensis* (CHS) | BAA23373 |
| *Solanum tuberosum* (CHS) | AAB67734 |
| *Synechococcus* sp. (CHS-like) | CAE07508 |
| *Vitis riparia* (STS) | AAF00586 |
| *Vitis vinifera* (CHS) | BAA31259 |
| *Vitis vinifera* (STS) | P28343 |
| **Outgroup taxa** |  |
| *Arabidopsis thaliana* (KCS1) | NP195178 |
| *Brassica napus* (KCS1) | AAA96054 |
| *Dunaliella salina* (KCS) | AAK11266 |
| *Marchantia polymorpha* (KCS2) | AAO48425 |
| *Sinapis arvensis* (KCS1) | AAX58617 |
| *Zea mays* (KCS2) | CAC01441 |

**(b)**

| **Ingroup Taxa** | **Accession Number** |
| --- | --- |
| *Arabidopsis thaliana* | AAA82888 |
| *Dryopteris fragrans* | AHI15920 |
| *Fistulifera solaris* | GAX16907 |
| *Ginkgo biloba* | AMN10098 |
| *Glycine max* | ACN81820 |
| *Hibiscus cannabinus* | ADK24217 |
| *Ipomoea batatas* | BAG82851 |
| *Oryza sativa* | AAA69580 |
| *Oryza sativa* Japonica Group | CAA36850 |
| *Phaeodactylum tricornutum* CCAP1055 | XP002178079 |
| *Pinus taeda* | AGX45528 |
| *Streptomyces malaysiensis* | ATL83639 |
| *Thalassiosira pseudonana* CCMP1335 | XP002289936 |
| **Outgroup taxa** |  |
| *Luciola parvula* (luciferase) | AAC37253 |
| *Photinus pyralis* (luciferase) | CAA59281 |
| *Pyrocoelia miyako* (luciferase) | AAC37254 |
